# Aging Gut Microbiome in Healthy and Unhealthy Aging

**DOI:** 10.14336/AD.2024.0331

**Published:** 2024-03-31

**Authors:** Yangyanqiu Wang, Zhanbo Qu, Jian Chu, Shuwen Han

**Affiliations:** ^1^Huzhou Central Hospital, Affiliated Central Hospital Zhejiang University, Huzhou, Zhejiang, China.; ^2^State Key Laboratory of Complex Severe and Rare Diseases, Medical ICU, Peking Union Medical College Hospital, Peking Union Medical College & Chinese Academy of Medical Sciences, Beijing, China.; ^3^Key Laboratory of Multiomics Research and Clinical Transformation of Digestive Cancer, Huzhou, Zhejiang, China.; ^4^Fifth School of Clinical Medicine of Zhejiang Chinese Medical University (Huzhou Central Hospital), Zhejiang, China.

**Keywords:** aging, healthy, gut, micorbiome

## Abstract

The characteristics of human aging manifest in tissue and organ function decline, heightening susceptibility to age-related ailments, thereby presenting novel challenges to fostering and sustaining healthy longevity. In recent years, an abundance of research on human aging has surfaced. Intriguingly, evidence suggests a pervasive correlation among gut microbiota, bodily functions, and chronic diseases. From infancy to later stages of adulthood, healthy individuals witness dynamic shifts in gut microbiota composition. This microbial community is associated with tissue and organ function deterioration (e.g., brain, bones, muscles, immune system, vascular system) and heightened risk of age-related diseases. Thus, we present a narrative review of the aging gut microbiome in both healthy and unhealthy aging contexts. Additionally, we explore the potential for adjustments to physical health based on gut microbiome analysis and how targeting the gut microbiome can potentially slow down the aging process.

## 1. Introduction

With societal advancements, human global life expectancy has increased from 64.2 years in 1990 to 72.6 years in 2019, with projections indicating a further increase to 77.1 years by 2050 [1]. However, an extended lifespan does not automatically translate to healthy aging. As individuals age, there's a gradual decline in bodily functions, rendering them more vulnerable to various ailments. A growing number of age-related diseases begin to manifest [2]. Consequently, extensive research into aging has been undertaken. Interestingly, evidence indicates a connection between gut microbiota and aging [3].

All complex organisms are accompanied by intricate microbial ecosystems, and humans are no exception. The gut microbiota is an essential component of the human body, having established a symbiotic relationship with the human host through long-term coevolution [2]. As Nobel laureate Joshua Lederberg observed, humans and their symbiotic microorganisms constitute a superorganism. In 2010, the EU MetaHIT project published the human gut microbiome gene catalogue in "Nature," unveiling a total of 3.3 million genes from the human gut microbiota, which is approximately 150 times the number of human genes [4]. Further research indicates that 98% of the gut microbiota can be classified into four major phyla: *Bacteroidetes, Firmicutes, Proteobacteria,* and *Actinobacteria* [5].

As early as 1907, Nobel laureate Elie Metchnikoff proposed that certain gut microbial communities within the human body are related to health and longevity [6]. Through the analysis of extensive gut microbiome data, researchers have discovered that changes in the gut microbiome accompany the aging process throughout an individual's lifespan. A comprehensive assessment of gut microbiota in infants, adults, and elderly individuals revealed that *Bacteroides* and *Clostridium* species were dominated in infant gut microbiota, while *Escherichia coli* and *Bacteroides* species were more prevalent in the gut microbiota of elderly individuals. The *Bacteroidetes* to *Firmicutes* ratio during infancy, adulthood, and old age was 0.4, 10.9, and 0.6, respectively [7]. Low-error amplicon sequencing (LEA-seq) was used to track changes in the gut bacterial community over a 5-year period, uncovering that 60% of bacterial strains-maintained stability during this timeframe [8]. Drawing on the stability of the human gut microbiome, host gut microbiomes were categorized into "core microbiomes" and "variable microbiomes" [9-11]. While changes in core and variable microbiomes between individuals weren't significantly associated with chronological aging, the diminishment of diversity in the core microbiome correlated with human aging or accelerated age-related phenomena, such as "frailty and cognitive decline" [12-13].

**Figure 1.**
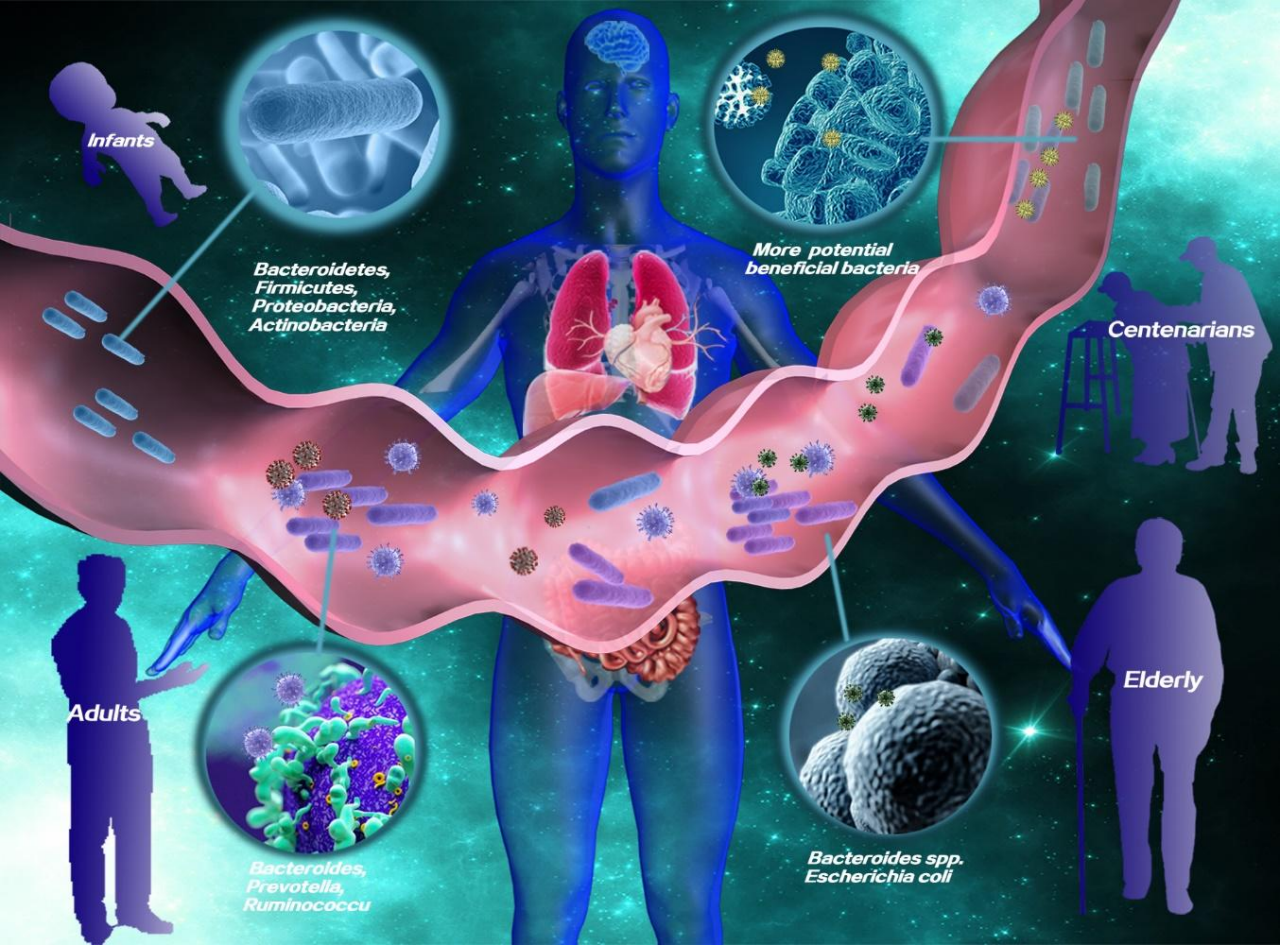
The graphical summary of aging gut microbiome in healthy and unhealthy aging

In 1992, Bocci introduced the concept of the gut microbiota as a metabolically active entity analogous to a virtual organ, labeling it the "neglected human organ." Over time, researchers have increasingly recognized the integral role of the gut microbiota in host physiology [14]. With the progress of multi-omics research in recent years, understanding the structure, diversity, and functions of the gut microbiome has emerged as crucial for establishing and sustaining health [15-18]. The development of the gut microbiota initiates from birth, possibly even earlier, facilitated by intricate mechanisms that support the proper development of the host organism. A 2012 study from Spain employed metatranscriptomic techniques to elucidate the functional roles of the host gut microbiome, highlighting families such as *Ruminococcaceae, Lachnospiraceae, Bacteroidaceae, Prevotellaceae,* and *Enterobacteriaceae* as major contributors to the functional microbial community in the gut. These families play vital roles in carbohydrate metabolism, energy conversion, and cellular component synthesis [19-21]. Metabolites produced by the gut microbiome, such as short-chain fatty acids, not only provide energy sources for the host but also regulate intestinal inflammation, promote vasodilation, influence specific gene expression including tumor suppressor genes, and modulate host metabolism [22-24]. Deviations from the norm in the structure and metabolic functions of the gut microbiota can lead to disturbances in the nervous, immune, and endocrine functions of both the digestive tract and distant organs. A tightly interwoven relationship exists between the gut microbiota and the human immune system, particularly the mucosal immune system of the gut. Reports suggest that the gut microbiota can modulate the generation of T cell populations in the host gut, such as "antigen-specific induced Treg cells" [27]. Moreover, the gut microbiome is implicated in the functioning of the neuroendocrine system, as observed in the gut-brain axis [25-26]. The gut microbiota and its metabolites not only directly affect intestinal function as signaling molecules but also indirectly impact the liver, brain, adipose tissue, and muscle, thereby influencing overall organism health. Alterations in the gut microbiota among elderly individuals are associated with specific age-related diseases (e.g., "frailty, decreased physical activity, cognitive decline, reduced bone density"). An imbalanced gut microbiota can trigger abnormal immune responses, increasing the risk of inflammatory gastrointestinal diseases or diabetes [28-32]. Consequently, maintaining a balanced gut microbial community is paramount for human health and longevity.

Our objective is to conduct a comprehensive examination of gut microbiota changes across various stages of life, focusing on both healthy aging and the perturbations observed in age-related diseases affecting extraintestinal organ axes (including the brain, heart, liver, pancreas, muscle, skin, and bones). We also intend to explore the potential anti-aging effects of interventions targeting the gut microbiota, such as dietary modifications, fecal transplantation, and microbiota-targeted therapies. We aspire that this review will provide guidance for further research into anti-aging strategies centered on gut microbiota modulation.

**Figure 2.**
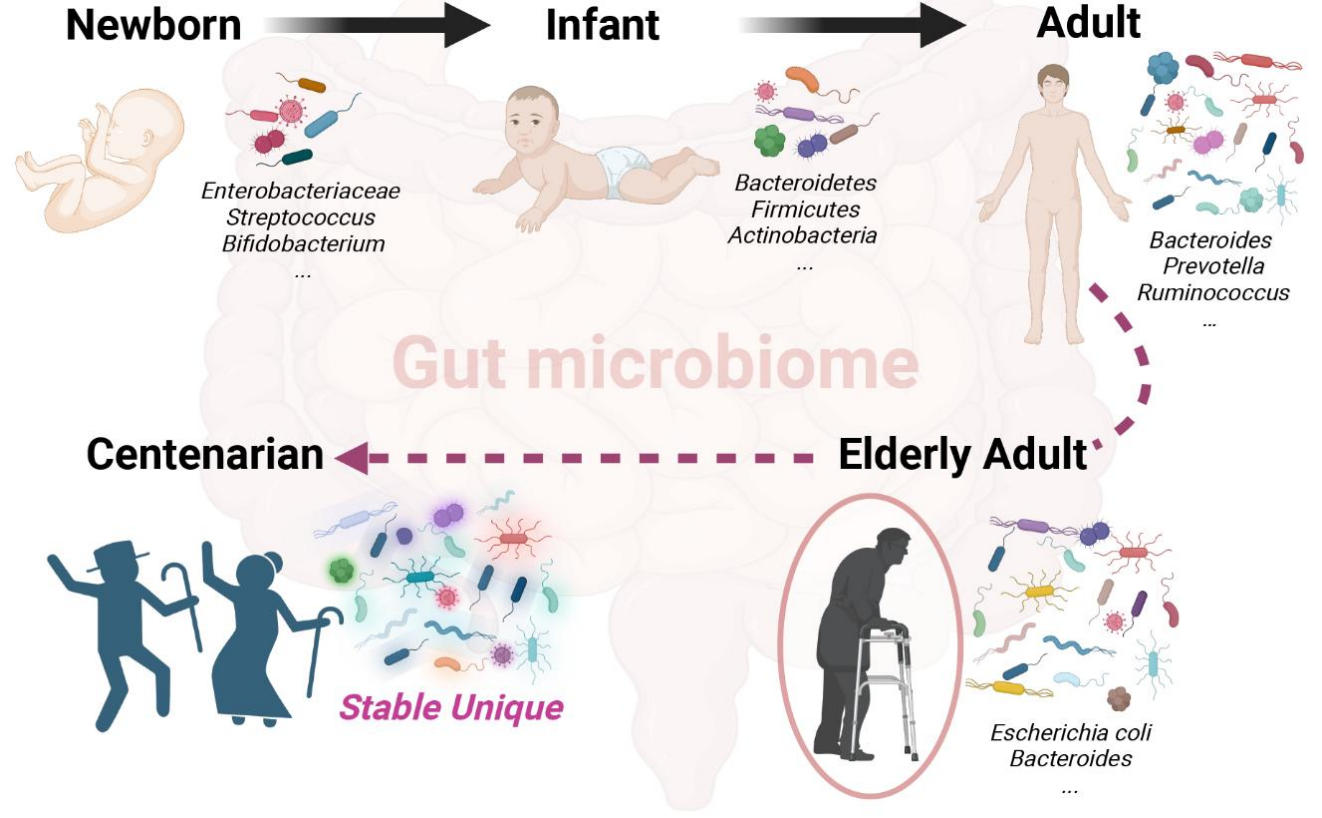
**Dynamic succession of gut microbiome during chronological ageing**. The gut microbiome undergoes shifts in dominant species throughout the whole life, with distinct core gut microbes identified for each stage. Interestingly, centenarians exhibit a gut microbiome distinguished by enduring stability and uniqueness.

## 2. Gut microbiome and healthy aging

### 2.1 Dynamic succession of gut microbiome during chronological aging

Throughout the human lifespan, changes and shifts in the gut microbiota accompany the aging process (Fig. 2). Infants undergo exposure to various environmental microbes from birth, leading to a gradual enrichment and increasing diversity of the gut microbiota [33]. Initial gut colonization in newborns primarily involves facultative anaerobic microbes like *Enterobacteriaceae* and *Streptococcus*, followed by obligate anaerobes such as *Bifidobacterium, Clostridium*, and *Bacteroides* [34-35]. By the age of 6 months, dominant phyla within the gut microbiota include *Bacteroidetes*, *Firmicutes, Proteobacteria,* and *Actinobacteria* [36]. Bacteria such as *Prausnitfaecali* and *mucin-loving Akkermansia muciniphila* were either absent or present at very low levels in early infancy and increased to adult levels around 1-2 years of age [37]. Influenced by both environmental and genetic factors [38-41], a symbiotic and stable gut microbiota typically forms in infants between 9 and 36 months of age [42]. This stable microbiota exhibits high bacterial diversity, with prevalent taxa including *Bacteroidetes, Firmicutes*, and *Actinobacteria* [43]. The gut microbiota composition in children aged 3-5 gradually converges towards that of adults. Once established, the gut microbiota's composition remains relatively stable throughout adulthood, although transient or persistent alterations can occur due to factors such as bacterial infections, antibiotic therapy, lifestyle, surgical interventions, diet, and physiological conditions [44]. Based on the core microbiome, the adult gut microbiota can be categorized into three enterotypes: *Bacteroides, Prevotella*, and *Ruminococcus* [45], representing diverse and stable states of the human gut microbiota. In middle-aged and elderly individuals, there is a decrease in gut microbiota diversity (e.g., "ELDERMET in Ireland and CENIT in Spain") [46-47]. Simultaneously, certain core gut microbial taxa undergo changes in older individuals. For instance, higher proportions of *Bacteroides spp.* and *Escherichia coli* have been observed, accompanied by reduced levels of *Faecalibacterium prausnitzii* in the gut microbiota of elderly participants [48-49]. The decrease in gut microbiota diversity and alterations in specific core microbial groups may serve as indicators of aging.

While research has provided an overall understanding of how the gut microbiota changes with age [50], it's worth noting that these studies lacked a longitudinal design. Discrepancies in gut microbial composition among individuals could stem from diverse factors including diet, antibiotic usage, living environment, and genetic variations. In forthcoming studies, the implementation of multi-generational longitudinal studies of fecal microbiomes holds promise for accurately tracking longitudinal shifts in the human gut microbiota throughout the lifespan.

### 2.2 Characteristics of gut microbiome in long-lived population

The gut microbiota changes with age. Centenarians appear to change more slowly than individuals below 100 years old. Research since 2010 has investigated the gut microbiota of elderly and centenarian cohorts from various regions including China (Guangxi, Hainan, Sichuan), Italy, Russia, and India. These studies have revealed that centenarians harbor a higher abundance of potential beneficial bacteria (such as *Bacteroides, Desulfovibrio suis, Pameliagodonibacterium pamelaeae, Ruminococcaceae, Lactobacillus, Akkermansia, Methanobrevibacter*) and a lower abundance of potentially harmful or pro-inflammatory bacteria (such as *Faecalibacterium, Prevotella, Klebsiella, Streptococcus, Enterobacter, Enterococcus*) compared to individuals below 100 years old [51-61]. The dominance of beneficial bacteria in the gut microbiota of centenarians might help counteract age-related health issues and aging. In 2023, a study on 297 centenarians from Guangxi, China, found that *Type 1 Bacteroides, Type 2 Shigella Escherichia, Type 3 Prevotella*, and *Type 4 Blautella* comprised 29.6%, 38.0%, 10.8%, and 21.6%, respectively, of the fecal samples of centenarians. The gut microbiota of centenarians displayed distinct and robust features, sharing a predominance of *Escherichia-Shigella* with elderly individuals compared to the predominance of *Bacteroides* in young individuals [61]. Additionally, there was an increase in species diversity and evenness in the gut microbiome of centenarians [51-61]. A 1.5-year follow-up study revealed an increase in overall α-diversity and Pielou index of the centenarian microbiota, accompanied by a decrease in richness, underscoring the importance of maintaining a balanced composition of different gut microbial communities for sustaining gut microbiota stability [61].

In 2019, an analysis of the gut microbial function in centenarians revealed heightened central metabolic capacities, particularly in glycolysis and fermentation pathways leading to the production of short-chain fatty acids. Additionally, centenarians also showed higher levels of phosphatidylinositol signaling systems, sphingolipid biosynthesis, and various levels of n -glycan biosynthesis [62-63]. In 2020, a functional investigation of the gut microbiome unveiled an increase in pathways associated with heterotrophic degradation metabolism and a decrease in pathways related to carbohydrate metabolism with advancing age [64]. Subsequently, a study conducted in 2021 analyzed the gut microbiota and metabolites of over 9,000 adults aged 18 to 101. Significant associations were identified between seven microbial metabolites, including indole and phenylacetylglutamine, and the distinct composition of gut microbiota in centenarians [65]. These two metabolites, previously shown to extend the lifespan of mice [66], were found at high levels in the blood of centenarians [67]. Furthermore, FADS1/2, PTPA, and FLVCR1 were found to be closely associated with arachidonic acid, succinylcarnitine, and choline, respectively, through gut microbiota and blood metabolomics analysis [49]. Notably, the fecal samples of centenarians were enriched with strains of *Rhizobiaceae* known to produce a bile acid derivative called isoalloLCA, which has demonstrated anti-inflammatory properties against *Clostridium difficile*-induced inflammation in mice [68]. In summary, the gut microbiota serves not only as a marker of aging but also plays a crucial role in maintaining human health and longevity.

## 3. Gut microbiome and Unhealthy aging

**Figure 3.**
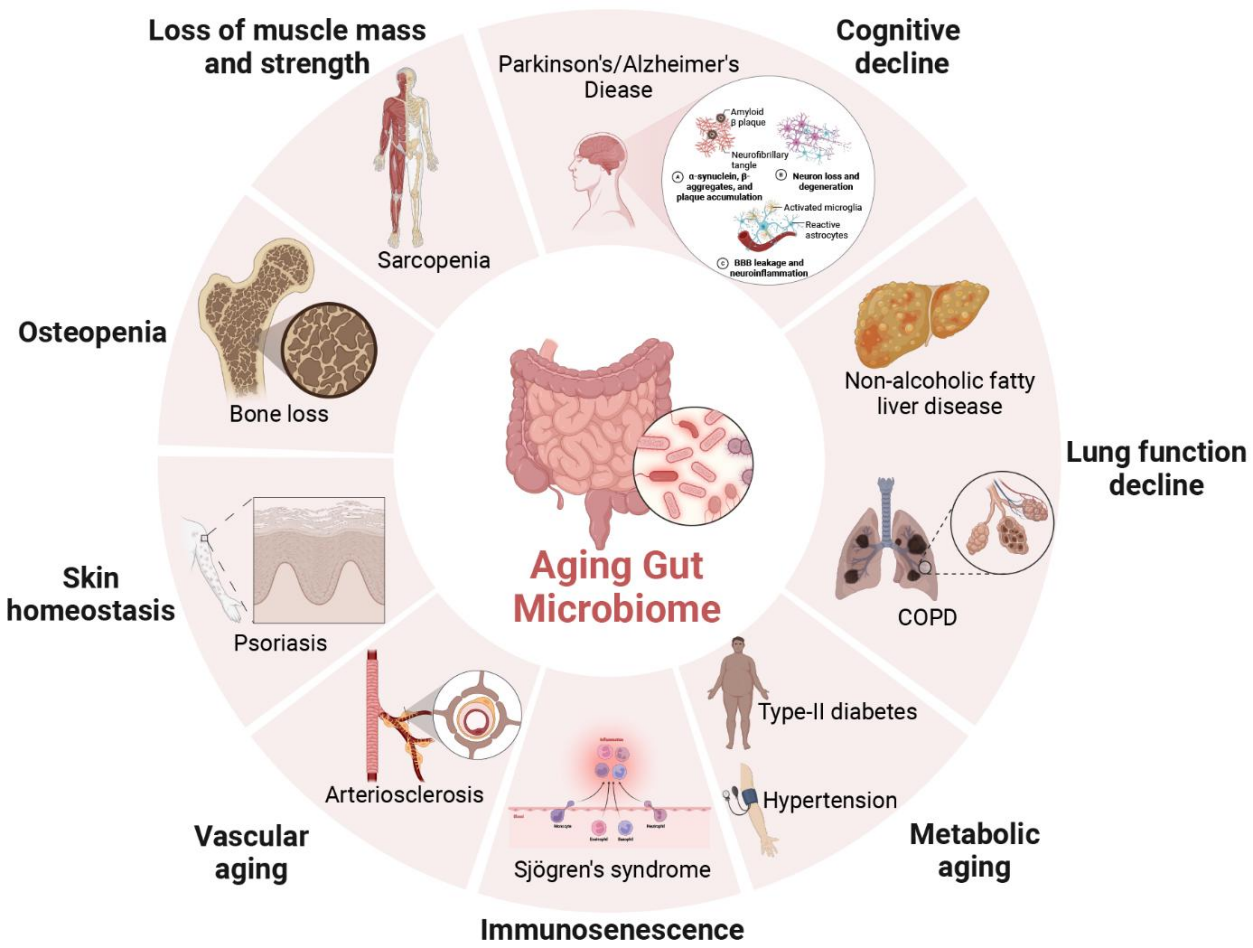
**The schematic of age-related gut microbiome associated with various organs and diseases**. Age-related changes in the gut microbiome are intricately linked with cognitive decline, decreased muscle mass and capacity, osteopenia, skin homeostasis, vascular ageing, immunosenescence, metabolic alterations, lung, and liver function.

From infancy to adulthood, our bodies become stronger. From adulthood to middle and old age, our bodies gradually become weaker. Frailty is a unique condition of the elderly, characterized by the gradual decline of physiological reserves, increased vulnerability of the body, decreased stress resistance, and multi-system dysfunction [69-71]. The age-related changes in the gut microbiota not only impact gut health but also extend to other physiological systems including the brain, bones, muscles, immune system, and vasculature (Fig. 3). The role of the gut microbiota in regulating various organ systems seems to be associated with frailty in older adults (Table 1). A comprehensive understanding of the gut microbiota's influence on various organ systems holds potential for enhancing the management and mitigation of the aging process.

### 3.1 Relationship between gut microbiome and cognitive decline in elder adults

The prevalence of age-related cognitive impairment and dementia is steadily increasing. Recent findings indicate a correlation between abnormalities in the gut microbiota and age-related cognitive decline [72-73]. A study examining fecal samples from healthy elderly individuals alongside brain electroencephalogram (event-related potentials, ERP) data provided initial evidence linking ERP outcomes with gut microbiota composition [74]. Analysis of cognitive performance and gut microbiota features revealed that physiological aging, encompassing cognitive and memory decline, is accompanied by changes in the abundance of certain bacteria such as *iron bacteria, Akkermansia, Muribaculaceae, Alistipes, Clostridium VadinBB60, Bacteroides, Faecalibacterium,* and *Actinobacteria* [75-77]. The gut microbiota is implicated as a potential key factor in age-related neurological disorders, including mild cognitive impairment, Alzheimer's disease, and Parkinson's disease. Gut microbiota associated with mild cognitive impairment or Alzheimer's disease include *Streptococcus thermophilus, Treponema denticola, Prevotella rumnicola, Tannerella forsythia, Eubacterium rectale*, and *Streptococcus intermedius* [78-81]. In Parkinson's disease, the dominated gut microbiota includes *Alistipes, Paraprevotella, Klebsiella, Sphingomonas*, and *Acinetobacter* [82]. The gut microbiota primarily influences human cognitive function through regulating microbial metabolites entering circulation and immune-inflammatory responses in the enteric and central nervous systems. Aging mice exhibit significant changes in the *Firmicutes/Bacteroidetes* ratio and the abundance of *Roseburia, Eubacterium,* and *Bifidobacterium* [83].

**Table 1:** Current findings on gut microbiome in unhealthy aging.

Disease/condition	Gut microbiome		Potential or known mechanism	Ref.
	Higher	Lower		
**Cognitive decline**	*Alistipes, Paraprevotella, Klebesiella, Sphingomonas, Acinetobacter, Aquabacterium, Desulfovibrio, Clostridium IV, Lachnospiracea*	*Lactobacillus, Sediminibacterium*	Metabolic effects; Neurological effects; Immune-inflammatory pathway	[82-83]
**Sarcopenia**	——	*Prausnitzii Faecalibacterium, Roseburia inulinivorans, Alistipes shahii* *Bacteroides, Bifidobacterium* *Roseburia, Clostridium*	Energy metabolism; Bile acid metabolism; systemic inflammation	[95-97] [103]
**Osteopenia**	*Clostridium difficile, Bacteroides,* *Escherichia coli*	*Collinsella, Megasphaera, Agathobaculum, Mediterraneibacter, Clostridium XIV,* *Dorea genera, Firmicutes/Bacteroidetes ratio*	Metabolic effects; Hormone regulatory pathway; Redox reaction	[107],[122]
**Skin ageing**	*Phyla Proteobacteria, Actinobacteria*	*Firmicutes*, *Bacteroidetes*, *Lactobacillus*	Metabolic effects; Redox reaction; Modulate Inflammatory	[118] [126]
**Vascular ageing**	*Desulfovibrio, Turicibacter*	*Lactobacillus, Bifidobacterium, Akkermansia*	Redox reaction; Metabolic effects	[127-129] [134-137]
**Immunosenescence**	*Firmicutes, Archaea, Proteobacteria*	*Bacteroidetes* *Bifidobacterium* *Lactobacillus helveticus* *Lactobacillus*	Inflammatory reaction	[143], [145], [147, 149]
**Metabolic ageing**	*Firmicutes Veillonellaceae*, *Enterobacteriaceae*, *Copri Prevotella*, *Bacteroides vulgatus*	*Bacteroidetes* *Akkermansia muciniphila*	Regulate lipid metabolism; Bile acid metabolism; Immune-inflammatory pathway	[149], [154]
**COPD**	*Prevotella*	*Streptococcus species, Micrococcacea*	Metabolic effects; Immune-inflammatory pathway	[162-163]
**NAFLD**	*Fissicatena_group, Romboutsia, Erysipelatoclostridium*	*Clostridia, Ruminococcaceae*	Regulate lipid metabolism	[169]

Additionally, plasma levels of phospholipid choline, oleic acid, linoleic acid, carnitine, pantothenic acid, and taurocholic acid are significantly decreased, while levels of propionic acid, hydroxybutyric acid, uric acid, and bile acids are significantly elevated. This suggests a correlation between gut microbiota aging and changes in plasma metabolites [84]. Gut microbiota aging in mice is associated with decreased short-chain fatty acids (SCFAs) in feces and impaired short-term and spatial memory [85]. Notably, both microbiota-aging mice and elderly individuals exhibit increased concentrations of trimethylamine N-oxide in blood and brain. The gut microbiota-derived metabolite delta-valerobetaine regulates age-related cognitive decline by modulating inhibitory synaptic transmission and neural network activity [86]. Another metabolite, isovaleramide (IAA), produced by *Ruminococcaceae* in the gut, induces microglial apoptosis by binding to the promoter region of the age-related gene S100A8, contributing to cognitive impairment in mice [87]. Furthermore, the gut microbiota can synthesize neurotransmitters (such as GABA, norepinephrine, and dopamine) and release neuroactive bacterial metabolites that regulate brain function by releasing hormones from enteroendocrine cells and dendritic cells, modulating immune and microglial cell function. Consequently, bacterial metabolites play a crucial role in aging and neurological disorders [88]. Moreover, Alzheimer's disease is characterized by inflammation around amyloid plaques, with specific gut microbiota subgroups (*Escherichia/Shigella, Pseudomonas, Rectalibacteraceae, Haemophilus, Clostridium,* and *Bacteroides*) potentially contributing to peripheral inflammation in patients with cognitive impairment and cerebral amyloidosis by increasing levels of pro-inflammatory cytokines (IL-6, CXCL2, NLRP3, and IL-1β) [89]. The increased activation of gut microbiota-inflammatory bodies correlates with enhanced astrocyte and microglia activation. Similarly, elevated expression of NLRP3 inflammasomes and IL-1β production is observed in the brains of 5xFAD mice, suggesting that the increased expression of inflammatory body components in the gut microbiota might be a crucial trigger for subsequent activation of downstream inflammation and potential cellular toxicity mediators [90]. In aging mice, plasma and brain lipopolysaccharide (LPS) levels are significantly elevated, accompanied by increased expressions of toll-like receptor 4 (TLR4) and myeloid differentiation factor 88 (MyD88) in the small intestine and brain. Gut-brain-axis dysfunction induced by LPS-induced TLR4/NF-κB signaling activation may contribute to age-related neuroinflammation and cognitive decline [91].

### 3.2 Relationship between gut microbiome and age-related loss of muscle mass and strength in elder adult

Sarcopenia poses a significant threat to the quality of life among elderly individuals, increasing the risk of mobility issues such as falls, fractures, and metabolic disorders, alongside increased hospital mortality rates. Studies comparing mice with intact gut microbiota and immune systems to germ-free (GF) mice lacking gut microbiota reveal that the latter exhibit reduced skeletal muscle weight [92]. Colonization of Duchenne muscular dystrophy mice with beneficial microbiota has shown promising results in reducing inflammation and improving muscle pathology and function [93-94], highlighting the potential role of the gut microbiota in the pathogenesis of sarcopenia. Human studies confirm a significant reduction in gut microbiota diversity in sarcopenia patients. Subjects with sarcopenia display distinct fecal microbiota composition at the species level, characterized by notable reductions in species known for their ability to produce short-chain fatty acids (SCFAs) such as *Prausnitzii Faecalibacterium*, *Roseburia inulinivorans*, and *Alistipes shahii* [95-96]. Additionally, reduced relative abundances of *Bacteroides* and *Bifidobacterium* genera in the aging gut microbiota are negatively correlated with muscle strength, while *Dolichum* and *Eggerthella lenta* are positively correlated with frailty [97]. Experiments in antibiotic-treated mice demonstrate decreased running endurance and lower anti-fatigue index in vitro, while transplanting a healthy gut microbiota result in increased muscle mass-to-weight ratio [98], underscoring the essential role of a healthy gut microbiota in optimal skeletal muscle function. Furthermore, the gut microbiota appears to play a crucial role in the skeletal muscle system by regulating energy metabolism, bile acid metabolism, systemic inflammation, and more. Gene deficiencies associated with SCFA synthesis, carotenoid and flavonoid bioconversion, and amino acid interconversion have been identified in the feces of sarcopenia subjects [99]. Additionally, alterations in key pathways such as riboflavin biosynthesis and energy metabolism contribute to skeletal muscle aging, with functional pathways related to transport proteins and biosynthesis of phenylalanine, tyrosine, and tryptophan being underrepresented in sarcopenia subjects, while lipopolysaccharide biosynthesis is overrepresented [100]. The downregulation of ileal FXR-FGF15/19 signaling in elderly male mice is attributed to changes in gut microbiota and microbial bile acid metabolism during the aging process. Activation of ileal FXR by fexaramine, a gut-specific FXR agonist, leads to increased skeletal muscle mass and improved muscle performance in elderly mice, with ileal FXR activation promoting skeletal muscle protein synthesis in an FGF15/19-dependent manner. In mice, ileal farnesoid X receptor (FXR)-fibroblast growth factor 15 (FGF15) signaling was inhibited by microbiota-mediated BA interference. Circulating FGF15 decreased, leading to downregulation of skeletal muscle protein synthesis via the extracellular signal-regulated kinase 1/2 (ERK1/2) pathway. Treatment with FGF19 (human FGF15 analog) partially reversed skeletal muscle loss in Abx mice [101-102]. Additionally, ghrelin-deficient (Ghrl-/-) mice exhibited characteristics of a pro-inflammatory gut microbiota profile, notably reduced bacteria producing butyrate salts, such as *Roseburia* and *Clostridium XIVb*. Fasting-induced muscle loss in Ghrl-/- mice exacerbated, characterized by decreased expression of muscle-regulating factor MyoD, increased expression of protein degradation marker MuRF1, and altered mitochondrial function. Acylated and unacylated ghrelin treatment significantly increased mitochondrial respiratory capacity in C2C12 muscle cells [103]. Modulation of the gut microbiota shows promise in improving frailty syndrome in elderly patients [104]. For example, increasing the presence of the probiotic *Lactobacillus plantarum* strain TWK10 not only enhances muscle strength in young mice but also prevents age-related muscle strength loss in elderly mice, accompanied by elevated muscle glycogen levels. TWK10 also alleviates age-related declines in learning and memory capacity and bone mass [105]. High-dose supplementation of leucine promotes a significant reduction in the *Firmicutes/Bacteroidetes* ratio and, through AMPKα/SIRT1/PGC-1α modulation, improves skeletal muscle health in aging mice [106].

### 3.3 Relationship between gut microbiome and osteopenia in the elderly

In osteoporosis patients, an elevated relative abundance of pathogenic bacteria such as *Clostridium difficile, Bacteroides*, and *Escherichia coli* is observed, while producers of short-chain fatty acids (SCFAs), including members of the *Collinsella, Megasphaera, Agathobaculum, Mediterraneibacter, Clostridium XIV*, and *Dorea* genera, are diminished [107]. Gut-bone axis regulators, such as Insulin-like Growth Factor 1 (IGF-1) [108], can be modulated by the gut microbiota. A clinical study conducted by Harvard Medical School and Massachusetts General Hospital unexpectedly revealed that the probiotic strain *Lactobacillus paracasei BL11*, along with its metabolites and synthetics including SCFAs and γ-aminobutyric acid, stimulates endogenous growth hormone release through the gut-brain axis. This stimulation leads to the generation of growth factor IGF-1 in the liver, which in turn promotes chondrocyte proliferation and increases synthesis of cartilage matrix collagen and proteoglycans [109]. Treatment with *Lactobacillus rhamnosus GG* (LGG) for bone homeostasis in mice results in increased levels of *spore-forming Clostridia*, which in turn induces production of butyrate in the gut and circulation [110]. Augmenting SCFA levels can restore serum skeletal regulators such as IGF-1 and bone mass levels [111]. The impact of the gut microbiota on bone tissue involves complex mechanisms, including the regulation of serum oxidative stress levels, osteoclast cytokine production, and hormone modulation. Studies indicate that gut microbiota α diversity decreases and the *Firmicutes/Bacteroidetes* ratio significantly decreases in a 22-month-old aged osteoporotic rat model [112]. Reduced levels of gut microbiota *bifidobacteria* and *Firmicutes/Bacteroidetes* ratio are associated with elevated levels of flavin monooxygenase-3 and trimethylamine N-oxide (TMAO). This leads to increased oxidative stress, further accelerating aging by increasing tumor necrosis factor-α levels in serum, decreasing Sirt6 expression in long bones, promoting nuclear factor kappa-B acetylation, and inducing excessive expression and activation of tissue protease k [113]. Germ colonization from children has been found to prevent declines in bone mass and strength. *Akkermansia muciniphila* from children's gut microbiota can correct bone metabolic imbalances through the secretion of extracellular vesicles, thus preventing osteoporosis. These nanovesicles enter and accumulate in bone tissue, mitigating the osteoporotic phenotype by enhancing osteogenic activity and inhibiting osteoclast formation [114]. In mice, *Lactobacillus reuteri* and *Lactobacillus plantarum* restrict bone loss by suppressing osteoclastogenic cytokines produced due to estrogen deficiency in bone marrow and the gut [115]. Gut metabolite urea A inhibits osteoclast genesis and age-related osteoporosis by enhancing bone marrow macrophage autophagy capability [116]. Oral administration of cinnamic acid has been shown to increase gut microbiota diversity, improve bone loss, and enhance bone indices, accompanied by increased BMP/TGFβ/Smad signaling [117]. Therefore, modulating the gut microbiota presents a potential avenue for regulating age-related bone loss.

### 3.4 Relationship between gut microbiome and skin aging in elder adult

Skin aging is an inevitable process resulting from physiological changes over time. Both the gut and the skin are exposed surfaces of the body to the external environment, sharing similar signaling pathways, neural innervation, and hosting a vast array of microbes. Distinct characteristics have been identified in the facial microbiota of older Chinese women compared to younger counterparts. For instance, older skin harbors a higher abundance of the *phyla Proteobacteria* and *Actinobacteria*, while younger skin is enriched with *Firmicutes* and *Bacteroidetes*. Younger skin exhibits greater microbial richness, with *Streptococcus, Rothia*, and *Bacillus* genera dominating [118]. Research into the relationship between skin microbiota and clinical parameters of skin aging, including pigmentation, wrinkles, and texture, has confirmed the significant influence of the microbiota on the aging process [119]. The gut microbiota plays a role in shaping the skin microbiota. Metabolic byproducts of the gut microbiota, such as short-chain fatty acids (SCFAs) - propionate, acetate, and butyrate, are believed to exert influence on key features of the skin microbiota, thereby affecting the skin's immune defense mechanisms [120-121]. For example, propionate produced by gut *Propionibacterium* has a profound antibacterial effect against the most common *community-acquired methicillin-resistant Staphylococcus aureus* (CA-MRSA) strain, USA300. *Staphylococcus epidermidis* and *Staphylococcus capitis* demonstrate resilience to SCFA exposure [121-122]. Metabolic products generated by gut *Clostridium difficile*, such as free phenols and p-cresol (biomarkers of gut microbiota dysbiosis), can enter the bloodstream and accumulate in the skin. In contrast, daily intake of *Bifidobacterium longum* and low galacto-oligosaccharide can lower serum total phenol levels produced by the gut microbiota, thereby enhancing skin health in healthy adult women [123]. Moreover, *Lactobacillus plantarum* HY7714 probiotics regulate the tight junctions of Caco-2 cells by upregulating the expression of genes encoding occludin-1 (OCL-1) and zonula occludens-1 (ZO-1). The HY7714 EPS effectively enhances the hydration capacity of UV-induced human dermal fibroblast HS68 cells by reducing the production of matrix metalloproteinases (MMPs) and reactive oxygen species (ROS) [124]. Dbn1 had the highest transcriptional upregulation in aged mouse dermis, with a twofold increase in Dbn1-expressing cells. Dbn1 heterozygous mice display impaired skin barrier function and hydration. Transplantation of gut microbiota from 5-week-old mice into 12-month-old aged mice enhances skin hydration capacity and thickens the stratum corneum in aged mice [125]. Additionally, the supernatant of *Lactobacillus fermentum GDMCC 61827* fermentation significantly reduces intracellular reactive oxygen species production and stabilizes mitochondrial membrane potential in UV-damaged skin cells, demonstrating anti-photoaging effects both in vitro and in vivo. In short, probiotics exhibit restorative effects on skin photoaging through antioxidation, reduced extracellular matrix degradation, and suppressed inflammatory factor expression [126]. These findings underscore the close association between gut microbiota and skin aging, suggesting that targeting the gut microbiota could potentially improve the state of skin aging.

### 3.5 Relationship between gut microbiome and vascular aging in elder adult

Similar to a person's actual age, blood vessels also have a corresponding age. Vascular age refers to the functional state of human arteries, especially large arterial vessels. Currently, the number of sudden deaths is increasing every year, with a significant portion attributed to cardiovascular diseases, most of which are caused by vascular functional disorders, suboptimal vascular health, and vascular diseases. Symbiotic microbiota of the halobiont, including *Lactobacillus, Bifidobacterium*, and *Akkermansia*, plays a pivotal role in maintaining vascular function and structural homeostasis [127-129]. Vascular remodeling in germ-free mice exhibits distinct changes, characterized by reduced resistance to arterial constriction, increased vascular stiffness, outward hypertrophic remodeling, and chronic reduction in blood flow [127]. Oxidative stress-mediated arterial dysfunction, such as endothelial dysfunction and increased stiffness of large elastic arteries, constitutes a major driver of age-related cardiovascular diseases. The gut microbiota intricately regulates host-wide oxidative stress. Animal studies have shown that inhibiting the gut microbiota via broad-spectrum, poorly absorbable antibiotics results in improved vascular endothelial function after 3-4 weeks [130]. Plasma concentrations of trimethylamine N-oxide (TMAO), a gut microbiota-derived metabolite, escalate with advancing age. Healthy older individuals exhibit higher plasma TMAO levels (6.3±5.8 μM) compared to their younger counterparts (1.8±1.4 μM) (p<0.001). TMAO positively correlates with carotid-femoral (c-f) or aortic (a) pulse wave velocity and systolic blood pressure. Dietary supplementation of TMAO increased aortic pulse wave velocity (aPWV) in young mice and exacerbated the elevated aPWV in aged mice, both groups experiencing an increase in systolic blood pressure by ~10 mmHg [131]. In young mice, adding trimethylamine N-oxide to the diet for 6 months caused aging-like damage to carotid artery endothelial-dependent acetylcholine dilation compared to the control group. This damage was accompanied by increased vascular nitrotyrosine, a marker of oxidative stress, which was reversed by superoxide dismutase mimic 4-hydroxy-2,2,6,6-tetramethylpiperidine-1-oxyl [132]. In healthy middle-aged/older individuals, higher plasma trimethylamine N-oxide levels correlated with higher nitrotyrosine content in endothelial cells on biopsy. Ascorbate infusion, an antioxidant, restored flow-mediated dilation to youthful levels, suggesting that high circulating trimethylamine N-oxide suppresses oxidative stress-related endothelial function by strong compensatory oxidative stress inhibition [132]. Prolonged exposure to oxidative trimethylamine N-oxide induces senescence in human umbilical vein endothelial cells (HUVECs), characterized by reduced cell proliferation, increased expression of senescence markers, G0/G1 arrest, and impaired cell migration. Additionally, TMAO inhibits SIRT1 expression, increases both in vivo and in vitro oxidative stress, leading to activation of the p53/p21/Rb pathway and reduced phosphorylation of CDK2, cyclinE1, and Rb [133]. Supplementation with gut microbiota metabolites such as DMB, ketones, resorcinol, and polyphenols promote the relative abundance of beneficial microbes (*Akkermansia* and *Lactobacillus*) while diminishing putative pro-inflammatory taxa (*Desulfovibrio* and *Turicibacter*), thereby exerting a beneficial effect on cardiovascular function [134-137].

### 3.6 Relationship between gut microbiome and immunosenescence in elder adult

Immune aging, a progressive phenomenon characterized by immune dysregulation, involves the restructuring of lymphoid organs and subsequent alterations in immune function in elderly individuals. These alterations include thymic atrophy, heightened differentiation of T cells, diminished proliferative capacity of T cells, reduced natural killer (NK) cell cytotoxicity, and compromised quantity and quality of antibody production by B cells [138-140]. The complex process of immune aging is regulated by factors such as chronological age, chronic inflammation, and microenvironmental changes, including those related to the gut microbiota [141-142]. For instance, age-related shifts in gut microbiota composition, marked by a decline in Bacteroidetes abundance, are associated with immunosenescence in middle-aged and elderly individuals. The presence of *Bacteroidetes* is positively correlated with serum immunoglobulin G (IgG) levels and the percentage of CD8+ T cells, and negatively correlated with the CD4+/CD8+ ratio. Conversely, *Firmicutes* presence negatively correlates with IgM levels, and the *Bacteroidetes* to *Firmicutes* ratio positively correlates with IgG and IgM levels [143]. Increasing the *Firmicutes* to *Bacteroidetes* ratio in the gut of mice upregulates the expression of Bim and FOXO3 in Foxp3+ regulatory T cells (Tregs), concurrently reducing the overall number of CD3+ T cells and naive T cells [144]. Furthermore, the abundance of *archaea* and *Proteobacteria* in the gut microbiota of aged animals surpasses that of young adults, with their levels directly correlating with plasma biomarkers indicative of inflammation and immune activation, such as neopterin, CRP, TNF, IL-2, IL-6, IL-8, and IFN-γ [145]. Age-related changes in gut microbiota composition, coupled with declining gut tissue function, may trigger innate immune responses and chronic inflammation, contributing to various age-related degenerative diseases and suboptimal aging outcomes. These include diminished vaccine efficacy, heightened susceptibility to viral and bacterial infections, as well as increased incidence of cancer and rheumatic diseases. Consequently, modulating the gut microbiota holds promise for partially restoring cellular immune function [146]. For instance, supplementation with a mixture of *Bifidobacterium longum Bar33* and *Lactobacillus helveticus Bar13* has been shown to bolster immunity in elderly individuals (aged 75 and older) and aged mice. This supplementation leads to increased levels of regulatory T cells (Treg and Tr1), reduced γδ T cells, and enhanced B cell populations in the bloodstream [147]. Additionally, supplementation with *Lactobacillus HN019* enhances the ex vivo phagocytic capacity of mononuclear and polymorphonuclear phagocytes, as well as the cytotoxic activity of natural killer cells in elderly subjects [148].

### 3.7 Relationship between gut microbiome and metabolic aging in elder adult

As individuals age, metabolic capacity declines, rendering the body more vulnerable to conditions such as hypertension, hyperlipidemia, and hyperglycemia. The gut microbiota, a pivotal component of human metabolism, undergoes changes in both composition and quantity, exerting profound effects on metabolic functions [149-155]. The phylum *Bacteroidetes S24-7* and *Firmicutes Veillonellaceae* have been linked to hypertension [149]. Metabolites derived from the gut microbiota, such as trimethylamine N-oxide (TMAO), have been shown to induce arterial stiffness and elevated systolic blood pressure in both mice and humans as they age [131]. The gut microbiota regulates energy provision and fat absorption for the body by modulating enzymes and regulatory factors crucial for lipid metabolism. Lactic acid produced by *Bifidobacterium* inhibits intestinal cell chylomicron secretion and promotes lipid storage by converting absorbed lactic acid into acetyl coenzyme A, subsequently inhibiting lipid beta-oxidation. Similarly, acetic acid produced by *Escherichia coli* inhibits intestinal cell chylomicron secretion while promoting lipid oxidation, ultimately upregulating the AMPK/PGC-1α/PPARα pathway [150]. Moreover, the gut microbiota modulates bile acid synthesis and metabolism in the liver. By activating the FXR receptor and reducing tauro-β-muricholic acid levels, the gut microbiota promotes the upregulation of the Fgf15 factor by the FXR receptor, leading to the inhibition of the classic bile acid synthesis pathway involving cytochrome P450 family 7 subfamily B member 1. This regulatory mechanism enables the gut microbiota to reduce secondary bile acid formation, regulate bile acid synthesis, secretion, and excretion in the liver, and consequently lower liver cholesterol levels [151]. In addition, the gut microbiota influences the body's blood glucose levels. Bacterial flagellin proteins from gut-derived bacteria, including *Enterobacteriaceae*, induce inflammation and dysfunction of pancreatic beta cells. This inflammatory response, mediated by toll-like receptor (TLR)-5 expressed on resident islet macrophages, is associated with beta cell dysfunction characterized by reduced insulin gene expression, impaired insulin processing, and stress-induced hypersecretion of insulin [152]. Furthermore, insulin sensitivity abnormalities play a pivotal role in disruptions of glucose and lipid metabolism within the body. Reduced production of the gut microbiota metabolite butyrate, due to the absence of the commensal bacterium *Akkermansia muciniphila*, impairs gut integrity, leading to leakage of bacterial products such as endotoxin. This activates CCR2+ monocytes, which infiltrate the retina and convert B1a cells to 4BL cells, inducing insulin resistance in aged mice by expressing 4-1BBL [153]. *Prevotella copri* and *Bacteroides vulgatus* are identified as major species driving the association between branched-chain amino acid biosynthesis and insulin resistance. *Prevotella copri*, in particular, induces insulin resistance and exacerbates glucose intolerance [154]. Compounds such as nopaline and Sargassum fusiform fucoidan reshape the gut microbiota and contribute to lowering blood glucose through the gut-metabolism axis [155-156].

### 3.8 Relationship between gut microbiome and physical organ in elder adult

#### 3.8.1 Age-related lung disease

The elderly face an increased risk of respiratory system diseases, such as chronic obstructive pulmonary disease (COPD), asthma, idiopathic pulmonary fibrosis, or lung infections [157]. Concurrently, patients with chronic lung diseases, including asthma and COPD, often experience inflammatory bowel diseases (IBD) and other gastrointestinal disorders [158-161]. The abundance of gut microbiota in COPD patients was significantly lower than that in healthy individuals. Predominantly, COPD patients exhibit a gut microbiota type dominated by *Prevotella*, alongside lower levels of short-chain fatty acids in the intestines [162]. Additionally, certain *Streptococcus* species and the family *Micrococcaceae* in the gut were correlated with reduced lung function [163]. Thirteen metabolites produced by the gut microbiota, including N-acetylglutamate and N-amino-formylglutamate, are related to reduced gastrointestinal function in COPD patients [163]. The gut microbiota diversity decreases with age, leading to an increase in pro-inflammatory factors. This inflammation can disrupt the cell structure of the intestinal lining, impairing its protective barrier function. Simultaneously, changes in the gut microbiota can affect the metabolism of substances like caffeine in the lungs, heightening susceptibility to pathogenic microorganisms [164]. Furthermore, gut microbiota imbalance might regulate the lung immune system. For instance, the genus *Fusobacterium* promotes the migration of group 2 innate lymphoid cells (ILC2s) from the intestine to the lungs and induces the production of IL-33 [165]. Increased production of microbial endotoxins activates the TLR4/NF-kB signaling pathway, triggering lung oxidative stress [166]. Co-culturing commensal bacteria like *Klebsiella pneumoniae* isolated from feces can reduce intestinal inflammation, enhance mitochondrial and ribosomal activity in colon cells, restore abnormal host amino acid metabolism in serum, thereby inhibiting lung inflammation and alleviating COPD [167]. Furthermore, Qibai Pingfei Capsule can modulate the gut microbiota composition in COPD rats, improving community structure, inhibiting the relative abundance of *Coprococcus_2, Prevotella_9*, and *Blautia* in the intestines, while increasing the relative abundance of gut *Prevotellaceae_UCG_003*. This treatment also improves lung function in COPD rats and promotes a new balance between Th17 and Treg cells. Modulating the gut microbiota may offer a beneficial approach to regulating age-related lung diseases [168].

#### 3.8.2 Age-related liver disease

Non-alcoholic fatty liver disease (NAFLD) is highly prevalent among the aging population and is characterized by liver lipid accumulation. As NAFLD progresses, there is a observed decrease in the abundance of *Clostridia* and *Ruminococcaceae* in the gut, accompanied by an increase in inflammation-related microbes such as *Fissicatena_ group, Romboutsia,* and *Erysipelatoclostridium* [169]. MBG mice, gavaged with *Escherichia coli/Bacteroides* and provided a diet deficient in protein/fat, exhibited diffuse lipid deposition and significant alterations in fatty acid, glycerophospholipid, and retinol metabolism [170]. Exposure to specific gut microbiota can exacerbate liver function damage by increasing triglyceride accumulation, especially in the context of malnutrition, indicating the potential driving role of gut microbiota in NAFLD. The severity of NAFLD correlates with changes in metabolic functions of the gut microbiota, including carbohydrate, lipid, and amino acid metabolism [171]. Increasing the abundance of beneficial bacteria such as *Akkermansia, Romboutsia, norank_f_Bacteroidales_S24-7_group* and decreasing the abundance of pathogenic bacteria such as *Klebsiella, Anaerotruncus, Bacteroides, Lachno-spiraceae_UCG-001, Lachnospiraceae_ NK4A136_ group, Ruminiclostridium, uncultured_f__Rumino-coccaceae, Desulfovib*, may help reduce hepatic triglyceride (TG) content and serum low-density lipoprotein (LDL) levels in mice [172]. *Lycium barbarum* polysaccharides may restore gut microbiota by increasing the abundance of certain *Bacteroides* species, decreasing the ratio of *Fusobacterium* and the ratio of *Firmicutes/ Bacteroides*, and alleviate NAFLD through the hepatic lipopolysaccharide/TLR4/NF-κB signaling pathway [173]. The prebiotic *spirulina* can regulate the gut microbiota, such as increasing the proportion of *Roseburia* and *Lactobacillus*, thereby improving transaminase levels, hepatic steatosis, and hepatic inflammation [174].

In short, functional changes in brain, muscle, skin, blood vessels, immune system, etc. are accompanied by gut microbial alteration during aging. Mostly, gut microbiota gets involved in the aging process by secreting metabolites. The mechanisms by which gut microbiota functions in unhealthy aging are diverse and often involve local inflammatory response and decreased immune function. A potential causal relationship exists between gut microbial changes and unhealthy aging of multiple body organs. The regulation of gut microbiota in the body's anti-aging and anti-disease processes is expected to achieve the extension of life span. Accordingly, more research is warranted to discover more longevity-promoting microbiota and further explore the mechanisms between them and hosts.

**Figure 4.**
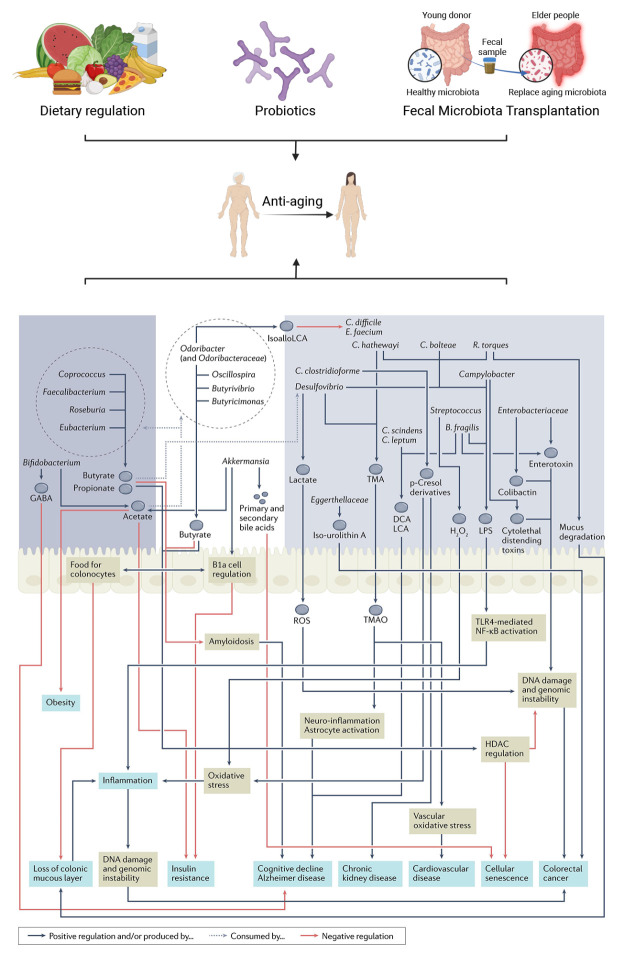
**Gut microbiota and specific commensals are potential anti-ageing agents**. Dietary interventions, probiotics, and fecal microbiota transplantation are among the strategies available to modulate the stability of the gut microbiota and exert anti-ageing effects. This figure partially comes from a reference entitled “The gut microbiome as a modulator of healthy ageing”. Metabolic capabilities of the three taxa groups are linked to unhealthy ageing-linked decline in host physiology. Graphic summary of the key metabolites or effectors produced by the three taxa groups and the effect each of these microbiome-derived entities has in either negatively or positively regulating various ageing-linked diseases and disorders. DCA, deoxycholic acid; HDAC, histone deacetylase; IsoalloLCA, isoallolithocholic acid; LCA, lithocholic acid; LPS, lipopolysaccharide; p-Cresol, para-cresol; ROS, reactive oxygen species; TMA, trimethylamine; TMAO, trimethylamine-N-oxide.

## 4. The role of enterovirus in aging

Intestinal viruses, including bacteriophages and viruses infecting gut bacteria, constitute essential components of the gut microbiota. However, while there has been extensive research on gut bacterial communities, investigations into the association between gut viral communities and aging remain limited. A recent study suggests that aging may correlate with a decrease in gut viral abundance and an increase in specific types of gut viruses, such as *CrAss-like phages*. Comparing virus genome data from two cohorts—individuals aged 60-100 (elderly group) and individuals aged 18-60 (adult group)—researchers found that centenarians have a more diverse range of gut viruses, including numerous previously unidentified virus sequences, some of which are associated with *Clostridium bacteria.* Additionally, specific types of gut viruses in centenarians were found to enhance the breakdown of sulfates by gut bacteria. The microbiomes of centenarians showed increased potential for converting methionine to homocysteine, sulfates to sulfides, and taurine to sulfides, which aids in preserving the integrity of the gut mucosal barrier and sequestering bacteria and toxins [175]. Besides, individuals with increased levels of gut viruses from the families *Caudovirales* and *Siphoviridae* perform better in tasks related to processing and verbal memory, whereas higher levels of *Microviridae* were associated with greater impairment in executive function. Transplanting microbiota from donors with elevated levels of specific tailless viruses (>90% from the *Siphoviridae* family) improved novel object recognition in mice and upregulated immediate early gene expression associated with memory in the prefrontal cortex. In fruit flies, the addition of *Lactococcus Siphoviridae phages* to their diet increased expression of memory-related brain genes and improved memory scores [176]. These findings suggest a potentially significant role for gut viruses in promoting healthy aging. Furthermore, the interactions and influences between gut bacteria and viruses may also play a crucial role in preventing age-related diseases.

## 5. Regulation of Aging Gut Microbiota

Maintaining gut microbiota homeostasis is pivotal for healthy aging and longevity. Achieving this goal involves employing dietary interventions, probiotics, and fecal microbiota transplantation to stabilize gut microbiota and exert anti-aging effects (Fig. 4).

### 5.1 Dietary regulation

The establishment of the adult gut microbiome commences during infancy and is influenced by several factors, including exposure to the maternal microbiome, mode of delivery, and early dietary exposures. Diet plays a pivotal role in shaping the abundance and function of specific bacterial species.

The consumption of a high-fiber diet can yield advantageous outcomes for host health, including the modulation of nutrient absorption or the production of short-chain fatty acids (SCFAs) [177]. The location within the colon where SCFAs derived from fiber are produced may play a crucial role in metabolic health. Individuals consuming more than 100 grams of fiber daily from a diet rich in vegetables, fruits, and nuts exhibited notably lower serum cholesterol levels and increased fecal SCFAs [178]. An exchange of diets between African Americans and rural South Africans, who traditionally consumed low-fiber and high-fiber diets, respectively, led to decreased rates of mucosal proliferation and colon inflammation (indicators of colon cancer risk) in the African American group. Meanwhile, the South Africans experienced reciprocal unfavorable changes in these measures [179].

The Mediterranean diet, known for its high intake of olive oil, legumes, whole grains, fruits, and vegetables, moderate consumption of fish and dairy, and limited meat intake, is widely acknowledged as a regimen effective in disease prevention and promoting healthy aging. The microbial changes regulated by the Mediterranean diet are associated with increased production of short-chain/branched-chain fatty acids and decreased levels of secondary bile acids, p-cresol, ethanol, and carbon dioxide production. The enriched gut microbiota resulting from the Mediterranean diet is positively correlated with several markers of reduced frailty and improved cognitive function, while being negatively correlated with inflammatory markers such as C-reactive protein and interleukin-17 [180]. Furthermore, research suggests a positive correlation between the Mediterranean diet and the prevention of muscle loss [181], as well as its potential to enhance glucose tolerance, prevent obesity, and alleviate intestinal inflammation [182].

### 5.2 Probiotics regulation

Preparations of microbiota, including prebiotics, probiotics, genetically engineered bacteria, among others, play a crucial role in regulating the gut microbial community and preventing age-related changes. For instance, aging animals exhibit a distinct microbiota composition characterized by an increased ratio of non-saccharolytic to saccharolytic bacteria and a lower abundance of β-galactosidase. However, supplementation with galacto-oligosaccharides can increase the abundance of specific saccharolytic bacteria (such as *Bacteroides* and *Lactobacillus*), elevate β-galactosidase levels, mitigate age-associated alterations in gut permeability, enhance mucin thickness, and reduce gut leakage [183]. In the nematode Caenorhabditis elegans, taurine has been shown to extend lifespan, reduce obesity and gut permeability, and enhance physical function [184]. Additionally, enrichment with probiotics like *Lactobacillus* leads to reduced carbohydrate metabolism (e.g., glucose), increased abundance of amino acids (such as creatine and dimethylglycine), and improvements in cognitive decline [185]. Supplementation with *Lactobacillus casei LC122* or *Bifidobacterium longum BL986* has been found to ameliorate peripheral tissue oxidative stress and inflammatory responses in mice, increase hippocampal neurodegeneration and neurotrophic factor expression, and enhance learning and memory abilities [186]. *Clostridium butyricum MIYAIRI 588* extends nematode lifespan by modulating the insulin/IGF-1 signaling pathway and Nrf2 transcription factor levels [187]. *Fermented Lactobacillus JDFM216* promotes nematode lifespan and immune responses through nuclear hormone receptor activation [188]. *Escherichia coli* mutants enhance secretion of bacterial capsular polysaccharide and regulate host mitochondrial dynamics and the unfolded protein response (UPRmt). Purified bacterial capsular polysaccharide promotes longevity through ATFS-1 activation (a host UPRmt response transcription factor) [189].

From a human perspective, oral probiotics have been found to mitigate several indicators of skin aging, including acidic skin pH, oxidative stress, photodamage, and skin barrier dysfunction [190]. Notably, bacteria like *Lactobacillus* and *Bifidobacterium*, which engage with dermal fibroblasts in a photoprotective manner, exhibit anti-aging properties [191]. Moreover, prebiotic dextrins sourced from corn starch and lentil diets have been shown to stimulate the growth of *actinomycetes* and *bacilli* while reducing the prevalence of *thick-walled bacilli*, thereby offering benefits to overweight and obese children [192-194]. Additionally, *Cyclocarya paliurus* demonstrates the capacity to enhance metabolic function and alleviate chronic inflammation by augmenting the abundance of *Bifidobacterium anisopliae* and suppressing the toll-like receptor 4-mitogen-activated protein kinase (TLR4-MAPK) signaling pathway [195]. In conclusion, *Lactobacillus* and *Bifidobacterium* strains are the most popular probiotics, and combinations of multiple probiotics have good prospects for application.

### 5.3 Fecal Microbiota Transplantation

Fecal Microbiota Transplantation (FMT) holds the capability to transfer the entire gut microbial community, preserving species-level composition, thereby facilitating the restoration of age-related alterations. Demonstrating promise in addressing conditions like *C. difficile* infections and irritable bowel syndrome, particularly in cases marked by gut ecological dysregulation.

Increased abundance of *Proteobacteria* and *Cyanobacteria* and decreased abundance of *Verrucomicrobia* in the gut microbiota of were found in a premature aging mouse model. Transplanting the gut microbiota from healthy mice into premature aging mice resulted in improved health and lifespan. Notably, individual transplantation of *Verrucomicrobia* also extended the lifespan of premature aging mice [196]. Another study conducted fecal microbiota transplantation from long-lived individuals (aged 101 years) and ordinary elderly individuals (aged 70 years) into germ-free mice. Mice receiving gut microbiota from long-lived individuals exhibited longer small intestinal villi, lower accumulation of lipofuscin and β-galactosidase (markers of aging) compared to mice in the ordinary elderly group. Additionally, the gut microbiota of mice from the long-lived group exhibited higher α-diversity, higher abundance of *Lactobacillus, Bifidobacterium*, and short-chain fatty acid-producing bacteria [197]. Through fecal microbiota transplantation, aged mice partially restored peripheral immunity (especially in mesenteric lymph node immune cells) and improved defects in hippocampal microglia. Beneficial changes occurred in the mouse hippocampal metabolome (including vitamin A, GABA, Neu5Gc, arginine, and related pathways) and glutamine synthetase expression, leading to improvements in age-related memory, learning, and behavioral deficits [198]. Clinical trial reports suggest that FMT holds promise as a treatment option for neurodegenerative diseases [199] and may be effective in managing or preventing metabolic-related disorders [200], T2DM [201], and NAFLD [202].

However, the implementation of FMT is not that simple, as it involves the transplantation of human tissues, which could potentially lead to the spread of infections, including prions and other infections. Therefore, in addition to the ethical issues that need to be considered, screening for FMT is laborious, time-consuming, and costly. FMT requires strict control over the process and the research.

## 6. Concluding remarks: An exciting future ahead

This study conducts a systematic review of the aging gut microbiota in both healthy and unhealthy aging, proposing regulatory strategies to target the gut microbiota for the purpose of decelerating aging processes and enhancing overall health. Our findings underscore the significant advancements in current research on gut microbiota and aging, laying a robust groundwork for further investigation in this field. The gut microbiota represents a new avenue for studying healthy aging, and although aging is inevitable, a particular hallmark of healthy aging may be the balance between the gut microbiota, and thus maintaining a normal gut microbiota is a potential way to promote healthy aging. Despite substantial progress in elucidating the role and mechanisms of the gut microbiota in age-related intestinal and parenteral diseases (e.g., skin aging, bone loss, cognitive decline, etc.), delving into the more intricate mechanisms of gut microbiota action in aging warrants further exploration.

Constant stimulation of gut mucosal immunity by pathogenic gut microbes and unhealthy lifestyles perpetuates local and systemic inflammation. To gain a more precise comprehension, elucidating the intricate relationship between the gut ecosystem and gut mucosal immunity is imperative. Model organisms will spearhead investigations into the microbe-host interactions that contribute to senescence, leveraging their advantages such as short culture generations, abundant offspring, ease of laboratory maintenance and propagation, well-defined genetic backgrounds, and straightforward experimental manipulation, especially genetic manipulation, alongside phenotypic analyses. Exploring the specific functions of individual microbes will further expand researchers' understanding of the mechanisms through which gut flora shape the aging process.

The causative relationship between changes in the gut microbiota and aging remains uncertain. To address this ambiguity, comprehensive longitudinal studies involving sizable cohorts of healthy elderly individuals are imperative. Employing multi-omics methodologies in concert, these investigations seek to unveil the underlying mechanisms linking the gut microbiota to healthy aging. Such research endeavors will establish a standardized reference dataset for health, facilitating the elucidation of associations between the gut microbiota and age-related diseases, ultimately fostering the development of diagnostic frameworks for unhealthy aging. Nonetheless, delineating the composition of a healthy gut microbiota across the lifespan poses a significant challenge. Urgent actions are warranted to establish repositories comprising samples, microbial strains, multi-omics databases, and corresponding clinical datasets. These datasets should encompass diverse participants, accounting for familial genetics, lifestyle factors (diet, exercise, sleep, and antibiotic usage), and occupational settings. Furthermore, social dynamics, socioeconomic status, and regional economic disparities should be considered. Mere database creation is insufficient; there must be standardized inclusion criteria and sequencing methodologies. A large integrated database akin to the Cancer Genome Atlas Program (TCGA) could be established for global dissemination among researchers. Naturally, handling such vast datasets necessitates the involvement of proficient AI teams to effectively integrate and analyze the data using pertinent algorithms and technologies.

As research techniques in microbiome science evolve and refine, the focus has shifted from correlation-based studies to studies aimed at unraveling the mechanisms that enable the development of new therapeutic approaches centered on gut microbes. Consequently, specific strains and metabolites identified through research hold significant promise for modulating age-related bacteria to promote healthy aging. However, their effectiveness hinges on factors such as strain specificity, disease context, dosage, treatment duration, individual variabilities, and gut resilience. Despite their potential, the efficacy and safety of these strains and metabolites await validation through extensive clinical trials, and further exploration of their mechanisms of action is warranted. Through the integration of bacterial engineering techniques, researchers may develop safe and dependable microbial agents to foster healthy aging. Additionally, the study of relevant bacterial strains could serve as a key indicator for assessing human aging alongside other metrics (e.g., skin health, organ function), with the stabilization of gut microorganisms potentially serving as a fundamental measure of aging progression.
